# Improving Moderator Responsiveness in Online Peer Support Through Automated Triage

**DOI:** 10.2196/11410

**Published:** 2019-04-26

**Authors:** David N Milne, Kathryn L McCabe, Rafael A Calvo

**Affiliations:** 1 School of Information, Systems and Modelling Faculty of Engineering and Information Technology University of Technology, Sydney Sydney Australia; 2 School of Electrical and Information Engineering University of Sydney Sydney Australia; 3 Department of Psychiatry and Behavioral Sciences University of California (Davis) Davis, CA United States; 4 Medical Investigation of Neurodevelopmental Disorders Institute University of California (Davis) Davis, CA United States; 5 Dyson School of Design Engineering Imperial College London United Kingdom

**Keywords:** social support, triage, classification, natural language processing

## Abstract

**Background:**

Online peer support forums require oversight to ensure they remain safe and therapeutic. As online communities grow, they place a greater burden on their human moderators, which increases the likelihood that people at risk may be overlooked. This study evaluated the potential for machine learning to assist online peer support by directing moderators’ attention where it is most needed.

**Objective:**

This study aimed to evaluate the accuracy of an automated triage system and the extent to which it influences moderator behavior.

**Methods:**

A machine learning classifier was trained to prioritize forum messages as green, amber, red, or crisis depending on how urgently they require attention from a moderator. This was then launched as a set of widgets injected into a popular online peer support forum hosted by ReachOut.com, an Australian Web-based youth mental health service that aims to intervene early in the onset of mental health problems in young people. The accuracy of the system was evaluated using a holdout test set of manually prioritized messages. The impact on moderator behavior was measured as response ratio and response latency, that is, the proportion of messages that receive at least one reply from a moderator and how long it took for these replies to be made. These measures were compared across 3 periods: before launch, after an informal launch, and after a formal launch accompanied by training.

**Results:**

The algorithm achieved 84% f-measure in identifying content that required a moderator response. Between prelaunch and post-training periods, response ratios increased by 0.9, 4.4, and 10.5 percentage points for messages labelled as crisis, red, and green, respectively, but decreased by 5.0 percentage points for amber messages. Logistic regression indicated that the triage system was a significant contributor to response ratios for green, amber, and red messages, but not for crisis messages. Response latency was significantly reduced (*P*<.001), between the same periods, by factors of 80%, 80%, 77%, and 12% for crisis, red, amber, and green messages, respectively. Regression analysis indicated that the triage system made a significant and unique contribution to reducing the time taken to respond to green, amber, and red messages, but not to crisis messages, after accounting for moderator and community activity.

**Conclusions:**

The triage system was generally accurate, and moderators were largely in agreement with how messages were prioritized. It had a modest effect on response ratios, primarily because moderators were already more likely to respond to high priority content before the introduction of triage. However, it significantly and substantially reduced the time taken for moderators to respond to prioritized content. Further evaluations are needed to assess the impact of mistakes made by the triage algorithm and how changes to moderator responsiveness impact the well-being of forum members.

## Introduction

When facing tough times, often the best people to turn to are those who have been through similar challenges and who can provide empathy and support that is grounded in personal experience [1]. Asynchronous text-based forums are a common method for facilitating such peer support online and have been shown to reduce symptoms of distress [2] and improve one’s sense of empowerment [3]. Their online nature allows individuals to access help at any time, from any location, with minimal cost and effort [4]. They can often be accessed anonymously to mitigate the fear of stigma that can be a barrier to help-seeking, particularly among the young [5].

Although online communities have much to offer, there exist potential pitfalls. For instance, they often lack the involvement of mental health professionals; community members’ interactions may be influenced negatively by an individual’s current mental health status [6]; social difficulties may be exacerbated online because of missing social cues [7] or illness-related disinhibition or disorganization [8]. Furthermore, without appropriate oversight, risky and unsafe behaviors can emerge unchecked, such as the normalization of self-harm, [9] and there is some evidence that online peer support can be misused as a method of avoidance [10].

The involvement of mental health professionals and paraprofessionals (ie, trained volunteers) likely improves the safety and therapeutic value of online peer support [11-13]. Users of these online communities appear to be amenable to oversight; for example, Kummervold et al [14] obtained almost unanimous feedback that mental health professionals should actively participate in online conversations and/or provide passive safety monitoring. Outside of peer support, increased moderation of online communities in general has been shown to improve intention to participate [15] and the quality of contributions [16].

The key barriers to greater oversight of online peer support are cost and scalability. As these communities grow, they place a greater burden on their human caretakers, which increases the likelihood that people in need may be overlooked. To address these barriers, we described an automated triage system that aims to guide human moderators to the people whose messages most urgently require their attention. We evaluated the accuracy with which this system identifies urgent content and the extent to which it influences moderator behavior. The evaluation of accuracy was conducted using a dataset of manually prioritized forum messages. The evaluation of behavior change was conducted as a quasi-experimental time series analysis that tracked moderator behavior for several years before and one year following the introduction of the triage system.

These evaluations were made within the context of ReachOut.com, an Australian Web-based youth mental health service that aims to intervene early in the onset of mental health problems in young people. ReachOut.com is well known and popular among its target population. Approximately 1 in 3 young people in Australia are aware of the site [17], and in 2015 the website received about 1.58 million Australian visitors [18]. In a survey conducted in 2013, approximately 77% of visitors reported experiencing high or very high levels of psychological distress, which indicates that the site is reaching people in need [19]. The site hosts a lively peer support forum for those aged 14 to 25 years to seek help and share their experiences. This community is maintained by staff employed by the organization and young volunteers who are recruited and trained for the role. Staff and volunteers—collectively referred to as the Mod Squad—monitor posts and respond as needed with encouragement, compassion, and referrals to relevant resources. This study investigated how to ensure the moderation provided by these professionals and volunteers remains scalable.

## Methods

This section describes the triage system that was deployed and the method by which it was evaluated.

### The Triage System

The triage system automatically prioritizes each new forum message as belonging to one of the following 4 categories:

- *Green* indicates a message can be safely left for the community to address, without requiring intervention from a moderator. Most forum messages are expected to fall into this category.
- *Amber* indicates a message that is important, but not urgent. It is appropriate for the moderator to wait and see if the community will respond to it. If no response is forthcoming, then a moderator should intervene.
- *Red* indicates a message that should be responded to as soon as possible, likely because the author is in distress or the message content may be triggering to others.
- *Crisis* indicates the author or someone they know is at risk of harm. A moderator should respond as soon as possible and enact ReachOut.com’s existing escalation protocol if appropriate.

The triage system is embedded into the forum as a sidebar that provides a *to-do list* of crisis, red, and amber messages that have not received a response from a moderator. Additional widgets are also embedded below each forum message to display the priority assigned to it and whether it requires attention. Further details (including a screenshot) can be found in Multimedia Appendix 1.

The underlying algorithm that assigns these priorities relies on supervised machine learning, meaning that it learns automatically from examples of manually prioritized forum messages. The advantage of this approach—as opposed to manually specified rules—is that it can easily be adapted to new prioritization schemes or new online communities simply by feeding it new examples of prioritized messages. The algorithm can also easily be maintained by learning from any corrections it is given by moderators. Further details of the algorithm—and the features it relies on—can be found in Multimedia Appendix 2.

### Evaluation of Accuracy

The evaluation of accuracy was conducted using a test set of manually prioritized messages that were withheld from any training or tuning of the algorithm. Both training and testing data were sourced from the Computational Linguistics and Clinical Psychology (CLPsych) 2016 shared task [20], which provided 1227 messages (947 for training and 280 for testing) that were extracted from ReachOut.com forums and manually labelled (ie, given one of the 4 priority labels described above) by 3 independent annotators. A reliability analysis indicated that these annotators reached a Fleiss kappa of 0.706 and pairwise Cohen kappa scores ranging between 0.674 and 0.761, indicating that though the task is somewhat subjective, a reasonable level of inter-rater agreement was achieved.

The primary evaluation metric used was f-measure, or the harmonic mean of recall (ie, sensitivity) and precision (ie, positive predictive value). As this is a multiclass classification problem where rare classes (eg, red and crisis) are of greater interest, scores were macro-averaged across the classes, after excluding the majority class (ie, green). We also reported the algorithm’s performance in separating out content that is flagged for the moderators’ attention (ie, amber, red, or crisis) from content that can safely be left for the community to address (ie, green), and in separating urgent content (ie, red and crisis) from content that can safely wait (ie, green and amber). In both cases we reported f-measure of the minority class (ie, flagged or urgent). Interested readers are directed to Milne et al’s study [20] for more detailed information about the dataset and evaluation metrics.

### Evaluation of the Impact on Moderator Response Behavior

A quasi-experimental time series analysis was used to compare moderator behavior before and after the introduction of the triage system. The primary measures of interest were response ratio and response latency: that is, the proportion of messages that received at least 1 reply from a moderator and the time elapsed between a message being created and receiving its first reply from a moderator. The analysis considers only replies made by moderators to messages authored by peers (ie, ordinary community members). All messages made by other types of forum members (eg, trainee moderators and other affiliates) are ignored, as are messages made by moderators unless in response to a message from a peer.

These measures were compared across 3 distinct periods: *prelaunch*, *postlaunch*, and *post-training*. The prelaunch period captured moderator behavior before the introduction of the triage system—from July 19, 2012, to August 5, 2016 (ie, 1478 days). The postlaunch period captured the interim in which the triage system was available, but not accompanied by any guidance about how it should be used or how often it should be consulted. It extended from August 5, 2016, to November 27, 2016 (ie, 114 days). The post-training period captured moderator behavior when the triage system was fully integrated into their workflow, having been launched with a detailed training session for all moderators. It extended from November 27, 2016, to October 16, 2017 (ie, 323 days).

During these periods, all priorities (ie, green, amber, red, or crisis labels) were assigned automatically. During the postlaunch and post-training periods, they were assigned immediately as each message was created and immediately revealed to users of the triage system. For the prelaunch period, they were assigned retroactively, simply by running the triage algorithm over the previously collected messages. These priority labels were not revealed to the moderators until after the launch of the triage system, and thus were unable to influence their behavior during the prelaunch period.

The volume of content that required moderation varied over time and could potentially have a strong effect on moderator response behavior. To account for this variability, we constructed histograms for each message that captured activity levels during 5 hour–long periods starting 2 hours before the message was created, the hour that the message was posted, and ending 3 hours afterward. These histograms record the number of messages created and the number of unique authors separately for peers and moderators. Together these 4 histograms measure the load placed on moderators (ie, the level of activity of the forum) and the number of active moderators available to share that load.

For further details about how moderators were identified, how replies were tracked for messages, and how the activity histograms were constructed, please refer to Multimedia Appendix 3.

### Statistical Analysis

The triage algorithm was trained and evaluated using the scikit-learn Python Library, and the evaluation of moderator behavior was conducted using SPSS version 25 (IBM) statistical software for Mac. To assess whether the presence of the triage system and moderators’ training with it (ie, period) were significant factors for moderator response ratio, direct logistic regression was performed separately for each priority level (green, amber, red, and crisis), while also accounting for moderator and peer activity at and around the time the message was posted. The impact of the triage system and moderator’s training on moderator response latency was similarly evaluated using separate linear regression models for each priority level. Kolmogorov-Smirnov values and visual inspection indicated that response latency had a non-normal distribution, which was corrected using log transformation. Follow-up pairwise group differences were examined where significant overall tests were reported. Finally, to reduce the likelihood of type-I error, adjusted alpha levels were applied to account for multiple comparisons (*P*=.004).

### Ethical Approval

This research was approved by the Human Research Ethics Committee at the University of Sydney as protocol 2016/064.

## Results

### Algorithm Accuracy

Table 1 shows the performance of the triage algorithm using the dataset and official metrics provided by the CLPsych 2016 shared task [20]. The system outperformed previous task participants in macroaveraged f-measure, ranked third in separating flagged (ie, crisis, red, or amber) messages from unflagged (ie, green) messages, and fourth in separating urgent messages (ie, crisis or red) from nonurgent (ie, amber or green) ones.

### Volume and Severity of Messages

Table 2 provides a summary of the number of messages posted by peers within each evaluation period and how the algorithm automatically prioritized them. As expected, within each period (ie, each column), the majority of messages were green, and there were progressively fewer messages as priority increased.

**Figure 1:**
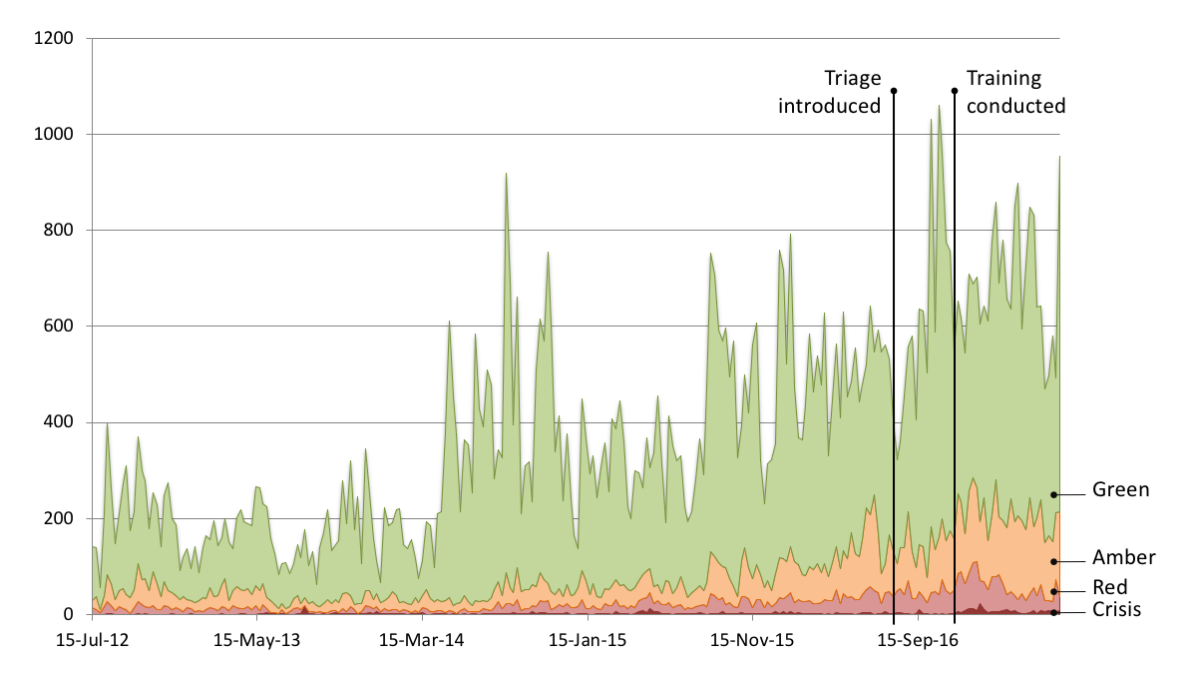
Weekly counts of prioritized messages from ordinary forum members.

Figure 1 provides a more detailed timeline, with a stacked histogram of the weekly volume of messages posted by peers, split into each of the 4 priorities. The number of messages posted each week fluctuated strongly, but there is a general upward trend indicating that the online community was becoming busier. At its peak, the forum received 1063 messages from peers during the week starting on July 2, 2017.

**Table 1:** Performance of the triage classifier and top participants of Computational Linguistics and Clinical Psychology (CLPsych) 2016.

Classification Algorithm	Macroaveraged *F*1	Flagged *F*1	Urgent *F*1
Kim et al [21]	0.42	0.85	0.62
Malmasi et al [22]	0.42	0.87	0.64
Brew [23]	0.42	0.78	0.69
Deployed to ReachOut.com	0.65	0.84	0.60

**Table 2:** Breakdown of how messages were prioritized (totals may be greater than 100% because of rounding).

Priority	Prelaunch, n (%)	Postlaunch, n (%)	Post-training, n (%)
Green	50,662 (80.1)	7808 (76.7)	22,008 (70)
Amber	8835 (14)	1634 (16)	6955 (22.1)
Red	3390 (5.4)	704 (6.9)	2170 (6.9)
Crisis	382 (0.6)	37 (0.4)	320 (1)

### Proportion of Messages That Received a Moderator Response

Table 3 provides a summary of how often messages from peers received at least 1 reply from a moderator. The data are organized by triage-assigned message priority, and the columns indicate whether the triage system was deployed at the time and whether training had been conducted.

Within each period (ie, column), there is a consistent progression in which the likelihood of a message receiving a reply is proportional to the priority assigned to it. The response ratios for green and red messages decrease after the introduction of triage but recover strongly after training. Response ratios for amber messages also decrease after the introduction of triage but do not recover after training. Response ratios for crisis messages change very little across the periods.

Logistic regression was performed to investigate whether these differences in response ratio were significant and to what extent they were because of the introduction of the triage system. Separate logistic regression models were completed for each priority label to predict the likelihood of a message receiving a moderator response. Each model contained 21 independent variables, including whether the triage system had been launched at the time of the message, whether training had been conducted, and the level of activity of moderators and community members at and around the time of the message (ie, the histogram data described previously).

For *green* messages, the model was significant (χ^2^_22_=11,411; N=80,478, *P*<.001), indicating that it was able to distinguish between messages that did and did not receive a moderator response. The model as a whole (ie, including all variables) explained between 13.3% (Cox and Snell R^2^) and 18.2% (Nagelkerke R^2^) of the variance in moderator response and correctly classified 70.2% of messages. In the final model (ie, retaining only statistically significant variables), the strongest predictor of moderator response was whether training for the triage system had been conducted, which recorded an odds ratio (OR) of 1.77. This was followed by the number of moderators online at the time of the community member message (OR 1.1). This indicated that green messages were more likely to receive a response after the triage system was introduced *and* moderators had been trained to use it after controlling for all other factors in the model.

For *amber* messages, the model was significant (χ^2^_22_=1207.2; N=17,424, *P*<.001). It explained between 6.7% (Cox and Snell R^2^) and 9.4% (Nagelkerke R^2^) of the variance in moderator response and correctly classified 70.3% of messages. The strongest predictor was whether triage training had been conducted recording an OR of 1.8. Again, this was followed by the number of moderators online at the time of the community member message (OR 1.3). This indicates that amber community messages were 1.8 times more likely to receive a moderator response after triage had been introduced, *and* moderators had been trained to use it after controlling for all other factors in the model.

For *red* messages, the model was again significant (χ^2^_22_=372; N=6264, *P*<.001), and explained between 5.8% (Cox and Snell R^2^) and 8.6 % (Nagelkerke R^2^) of the variance in moderator response and correctly classified 76.2% of messages. The strongest predictor of moderator response was the number of active moderators at the time the message was posted, recording an OR of 1.5. This indicated that messages that were posted when there were more moderators online were slightly more likely to receive a response, after controlling for all other factors in the model. The second strongest predictor of moderator response was whether training for the triage system had been conducted (OR 1.3).

Finally, for *crisis* messages, the model was also significant (χ^2^_22_=76.3; N=739, *P*<.001). It explained between 9.8% (Cox and Snell R^2^) and 15.8% (Nagelkerke R^2^) of the variance and correctly classified 81.2% of messages. The strongest predictor was the number of active moderators at the time the message was posted, recording an OR of 1.7, followed by the number of community posts at the time the message was posted. These were the only 2 variables to contribute significantly to the regression model. This indicates that crisis messages posted when there were more active moderators were almost twice as likely to receive a response after controlling for all other factors in the model. Neither the presence of the triage system nor training made statistically significant contributions to the model (*P*>.1).

**Table 3:** Proportion of messages that receive a reply from a moderator.

Priority	Prelaunch, n (%)	Postlaunch, n (%)	Post-training, n (%)
Green	17,004 (33.6)	1687 (21.6)	9699 (44.1)
Amber	5763 (65.2)	976 (59.7)	5322 (60.2)
Red	2568 (75.8)	461 (65.5)	1739 (80.1)
Crisis	308 (80.6)	30 (81.1)	261 (81.6)

**Table 4:** Time taken for moderators to respond to messages.

Priority	Prelaunch, median (IQR^a^)	Postlaunch, median (IQR)	Post-training, median (IQR)
Green	0:37:38 (10:24:35)	0:21:49 (6:54:12)	0:33:16 (10:35:09)
Amber	2:08:34 (11:33:11)	0:33:22 (4:05:39)	0:30:08 (4:48:54)
Red	2:09:45 (9:33:11)	0:42:17 (4:57:47)	0:26:25 (1:52:00)
Crisis	2:23:03 (7:23:54)	0:47:11 (1:40:34)	0:28:45 (1:15:50)

^a^IQR: interquartile range.

### Time Taken for Messages to Receive a Reply From a Moderator

Between prelaunch and postlaunch periods, the median time taken for moderators to respond to crisis messages was reduced from 2 hours 23 min to 47 min (see Table 4). Reductions were also observed for red (2 hours 10 min to 42 min), amber (2 hours 9 min to 33 min), and green messages (from 38 min to 22 min). Response latency also decreased between the postlaunch and post-training periods for crisis (from 47 min to 29 min), red (from 42 min to 26 min), and amber (from 33 min to 30 min) but increased for green messages (22 min to 33 min). Cumulatively, the presence of the triage system and the training of moderators resulted in reductions of 80%, 80%, 77%, and 12% for crisis, red, amber, and green messages, respectively.

Furthermore, the triage system reduced the variability of response latency for nongreen messages. The interquartile range (IQR) of latency for crisis, red, and amber messages was reduced by 77%, 48%, and 65%, respectively, between prelaunch and postlaunch periods. Between prelaunch and post-training periods, the same measures were reduced by 83%, 80%, and 58%, respectively. The IQR of latency for green messages decreased by 34% between prelaunch and postlaunch but remained steady (a 2% increase) between prelaunch and post-training.

A multiple regression analysis was used to further evaluate whether these differences in latency were statistically significant and whether they remained after accounting for moderator and community activity at and around the time of the message post (note: adjusted *P*=.004). Separate regression models were conducted for each priority level and included the same variables as the models for response ratio (described above).

For *green* messages, the total variance explained by the model as a whole was 47.9% (*F*_21,28368_=1243.7; *P*<.001). In the final model, the number of moderators online at the time the message was posted was the strongest predictor of moderator response latency (beta=−.33; *P*<.001), indicating that the more moderators online, the shorter the latency in response time. Whether or not moderators had been trained to use the triage system also contributed significantly to the model indicating that response time reduced significantly from prelaunch to postlaunch periods (beta=−.09; *P*<.001).

For *amber* messages, the total variance explained by the model as a whole was 18.8% (*F*_21,12039_=132.4; *P*<.001). Moderator activity during the hours before, at, and after a community member posted to the website, as well as triage period, were entered into the model. The number of active moderators at the time of the message made the strongest significant contribution to explaining moderator response latency (beta=−.35; *P*<.001). The presence of the triage system also made significant contribution to the model (beta=−.08; *P*<.001) indicating that response latency decreased from the prelaunch to postlaunch periods.

For *red* messages, the total variance explained by the model as a whole was 24.9% (*F*_21,4746_=76.2; *P*<.001). Moderator activity during the hours before, at, and after a community member posted to the website, as well as trial period, were entered into the model. In the final model, the number of active moderators at the time of the message made the strongest significant contribution (beta=−.40; *P*<.001), indicating that the more moderators online when a community member posted, the shorter the latency in response time. The presence of the triage system also made a statistically significant contribution (beta=−.14; *P*=.001) with response latency significantly decreasing from prelaunch to postlaunch periods.

For *crisis* messages, the total variance explained by the final model as a whole was 28.2% (*F*_21,577_=12.2; *P*<.001). The model consisted of moderator and community activity in the hours before, at, and after each message was posted. The strongest predictors of response latency were the number of active moderators at the time of the message (beta=−.34; *P*=.001), the number of moderator posts (beta=−.26; *P*=.001), and the number of active moderators in the hour preceding the message (beta=−.21; *P*=.003). This indicates that greater numbers of active moderators correspond to faster replies. Finally, the trial period also made a statistically significant contribution to the model (beta=−.17; *P*=.001).

To look more closely at the effect of triage period, planned comparisons with statistical correction (adjusted alpha=.004) comparing prelaunch and postlaunch periods (postlaunch and post-training) were conducted. These showed that messages were responded to more quickly during the post-training period (ie, combining the triage system with appropriate training) if they were labelled amber (*P*<.001) or red (*P*<.001). The large apparent difference in response latencies for crisis messages was significant only at the trend level (*P*=.007), likely because there were only 30 crisis messages in the postlaunch period.

Similar comparisons between postlaunch and post-training periods showed that messages were responded to more slowly in the post-training period if they were labelled green (*P*<.001) but more quickly if they were labelled red (*P*<.001), but the differences for amber and crisis messages were not significant (*P*>.05).

Finally, comparisons between prelaunch and post-training periods (ie, combining the triage system with appropriate training) showed that messages posted during the later period received replies significantly faster for all severity labels (*P*<.001).

## Discussion

### Summary of Findings

This study evaluated a triage system in terms of the accuracy with which it automatically prioritized content in online peer support and the extent to which it improved the responsiveness of human moderators to the prioritized content. The triage algorithm achieved high accuracy (84% f-measure) in identifying content requiring moderator response. Additionally, the combination of the triage system and appropriate training resulted in modest improvements to response ratio for all priority levels other than amber and large reductions to response latency for all priority levels other than green. Overall, the observed reductions in response latency and variability of response latency for flagged messages indicate that the triage system supported the online moderator as intended.

### Accuracy of the Triage Algorithm

Over the holdout test set, the triage algorithm was more accurate in separating out flagged (ie, nongreen) messages than it was in separating urgent (ie, red and crisis) messages. Arguably, the first of these boundaries is more important because this determines which messages enter the sidebar (see Multimedia Appendix 1). A low recall here could cause the moderators to miss posts that they should pay attention and respond to, whereas a low precision would increase their workload by filling the sidebar with low-priority messages. In contrast, mistakes made in separating urgent and nonurgent messages only effect the ranking of messages within this sidebar.

In addition to the above results, it was encouraging to observe a high level of agreement between the moderators and the triage algorithm during the prelaunch period (ie, the first column of Table 3), where there was a clear progression in which the likelihood of moderators responding to messages was proportional to the priority assigned by the algorithm. This agreement was in no way due to the triage algorithm influencing moderator behavior as, during severity, labels were assigned retroactively for the prelaunch period (ie, after the moderators’ responses had been made). Conversely, it was also in no way due to the moderators influencing the algorithm, as none of the features or training data used by the algorithm were based on moderator behavior. Thus, we are able to show that the moderators and the triage algorithm arrived at similar decisions independently.

### Impact on Moderator Response Ratio

As mentioned previously, the triage system resulted in only modest improvements to moderator response ratio. This is understandable, given that the moderators already prioritized responding to urgent messages before the introduction of the triage system. Evidence of this response hierarchy is seen in the clear progression within the prelaunch period (ie, the first column of Table 3), where crisis messages were more likely to receive replies than red messages which were responded to more often than amber messages which were in turn more likely to receive a response than green messages. The potential to improve response patterns after launch and/or training was limited because the moderators were already behaving as desired.

In fact, the introduction of the triage system appears to have initially had a detrimental effect, with response ratios dropping between prelaunch and postlaunch periods for all severity labels other than crisis. This may be because of the unfamiliarity of the system and the lack of training given during this initial postlaunch period. Fortunately, response ratios recovered in the post-training period, such that the end result (ie, between prelaunch and post-training periods) was an increase across all severity labels other than amber. The reduction in response ratio for amber messages is likely due to the way the triage interface allows these messages to be clearly marked as resolved when the community has rallied around them. In the post-training period, moderators were specifically instructed to only respond to amber messages if they had been overlooked by the community.

The strongest predictor for response ratio was whether or not moderators had been trained to use the triage system (for green and amber posts) or the number of moderators active at the time of the message (for red and crisis). It is important to note that the introduction of the triage system (with training) significantly increased the likelihood that green, amber, and red messages received a moderator response after accounting for moderator and community activity. The system was not a significant predictor for the response ratios of crisis messages.

### Impact on Moderator Response Latency

Before the introduction of the triage system, moderators took a median time of roughly 2 hours to respond to nongreen messages, whereas green messages tended to be responded to either quickly or not at all. The informal launch of the triage system led to large reductions in response latencies for amber, red, and crisis messages and modest reductions for green messages. The formal launch and associated training led to further reductions for amber, red, and crisis messages, and a substantial increase for green messages. Cumulatively (ie, between prelaunch and post-training periods), response ratios dropped by approximately 80% for crisis, red, and amber messages and 12% for green messages. Additionally, the variability (ie, IQR) of response rates dropped by approximately 60% for amber messages and approximately 80% for red and crisis messages but rose by 2% for green messages.

Across all priority levels, the strongest predictor of response latency was the number of moderators online at or around the time of the post. The formal launch of the triage system coincided with an influx of new volunteers, and their introduction had a large impact on response latency. However, it is important to note that the largest decreases in response latencies occurred between the prelaunch and postlaunch periods (ie, before this influx of new moderators). Additionally, the triage system and/or moderators’ training with it were shown to be a significant predictor for the reduced response latencies across all priority levels. Overall, the large reductions in response latency and variability of response latency for priority messages indicate that the triage system supports moderator behavior as intended.

### Limitations

This evaluation of moderator behavior has made a key assumption that the decisions made by the triage algorithm are correct and that it is desirable that moderators follow its recommendations. As mentioned above, the evaluation over the CLPsych 2016 dataset and the agreement observed between the moderators and the algorithm during the prelaunch period are encouraging. Nevertheless, it is important that future research evaluates the impact of the mistakes that any automated system will inevitably make, particularly given the inherent subjectivity of the prioritization task.

False negatives—messages that the system does not prioritize highly enough—have the potential to be particularly problematic. As moderators come to rely on the triage system, they may neglect to look elsewhere for high-priority content and consequently the system may counter-productively increase the chance that it is overlooked. We employed 2 strategies to decrease the likelihood of false negatives. The first was to reweight the triage algorithm so that it prioritizes recall ahead of precision, that is, it cares more about ensuring that any potentially urgent messages are included in the sidebar than ensuring that the sidebar includes only urgent messages. The second strategy was to deploy a tool that allowed ordinary community members to manually identify urgent content and add it to the triage system. The interface that enabled this crowdsourcing is described at the end of Multimedia Appendix 1. We would encourage other practitioners to provide similar safety nets if they adopt or develop an automated triage system.

Another limitation is that the analysis focused exclusively on the behavior of moderators and has not considered replies and support offered by community members, affiliates, or moderators in training. Moderators may systematically avoid responding to messages that receive a strong response from the community or encourage the community to be more self-reliant by withholding intervention when it is safe to do so. It is also possible that moderators will direct trainees to respond to urgent content rather than resolving it themselves. Our analysis has—necessarily, for the purposes of triage evaluation—assumed that a message is not resolved until it receives a reply from a moderator and that prompt replies are universally desirable. It would be more accurate to say that moderators should be kept aware of concerning content and be ready to intervene if necessary, rather than that they should always intervene as quickly as possible.

A related limitation is that the analysis has focused on moderator behavior without evaluating the impact this has on the community and its members. Although the underlying aim of increasing moderator responsiveness to urgent content has been to improve the safety and therapeutic value of the online community, we have not measured such outcomes directly. For future studies, it will be extremely valuable to survey forum users before and after the introduction of a triage system such as this to assess whether the changes to moderator behavior were noticeable and whether this leads to better outcomes or an improved sense of support.

A final, more technical limitation is that some of the variables considered by our statistical models are not entirely independent. Some of the variables introduced when modelling both response ratio and latency relate to the activity levels of moderators at and around the time of a message being posted. Intuitively, greater numbers of active moderators are likely to lead to more prompt responses. Unfortunately, moderator activity levels are not entirely independent from response ratio or latency, as there has to be at least 1 active moderator during one of these windows for a message to receive a reply. Consequently, these models may overestimate the impact of moderator activity. The same variables are also not entirely independent from the presence of the triage system, as it is possible that it has contributed to the activity levels of moderators by making the process of moderation more engaging. The combination of these 2 issues means that it is possible that these models have underestimated the individual impact of the triage system.

### Comparison With Prior Work

To our knowledge, this study is the first evaluation of automated triage in online peer support that has focused on behavior change, that is, the system’s ability to influence human moderators and direct their attention to where it is most needed.

There is, however, a great deal of prior work related to the machine learning and computational linguistics aspects of this study. This includes the CLPsych 2016 shared task [20] in which 15 teams of researchers competed to develop the best algorithm for prioritizing forum messages from ReachOut.com. Our classification system was directly informed by the submissions of the top performing teams [21-23] and was trained and evaluated on the same dataset. Encouragingly, researchers have continued to work with these data and hone their algorithms, with Cohan et al [24] significantly outperforming all previous participants. Additionally, a second edition of the shared task was run in 2017 and attracted 15 teams of researchers [25]. Thus, advances to the algorithm have already been made and are available for integration in future deployments of the triage system.

Also closely related are reports from Huh et al [26] and Delort et al [27] that describe machine learning systems to determine whether or not a forum message requires moderation. Both differ from our research by framing the problem as binary (a message either requires moderation or does not), whereas we have framed it as a multiclass prioritization problem. Both focus exclusively on algorithm accuracy, and do not investigate moderator behavior change.

In addition, there is a great deal of more broadly related work on the use of machine learning to detect undesirable content and behavior online. For example, researchers have developed algorithms to detect hate speech [28,29], cyberbullying [30-32], and the grooming activities of pedophiles [33]. There has also been much progress recently in applying natural language processing to social media to gain insights into the authors’ state of mind, or to identify and diagnose individuals who could benefit from some form of psychological intervention or assistance (see [34] for a recent review). For example, researchers have developed algorithms to detect suicide ideation [35-38], depression [39-41], post-traumatic stress disorder [41,42], and the *dark triad* of antisocial personality traits [43,44]. It is very likely that such algorithms could be applied successfully to triaging content in online peer support.

### Conclusions

This study has described a triage system that automatically prioritizes content in online peer support to augment human moderators and help them focus their efforts on the individuals and messages that have the greatest need. Through evaluation on a dataset of manually prioritized forum messages, we have shown that the triage algorithm is largely accurate, particularly for the critical boundary separating content that moderators need to pay attention to from content they can safely leave for peers to address on their own. Through a long-term field study, we have shown that the triage system greatly reduces the time taken for moderators to respond to prioritized content, and that moderators are largely in agreement with the triage system about which messages they should prioritize responding to.

Our underlying aim of this study in improving the responsiveness and scalability of human moderation in online peer support is to increase the safety and therapeutic value of these communities. However, further evaluations are needed to establish whether this is the case. Additionally, in the near future, we plan to investigate the impact of mistakes made by the triage algorithm and how best to encourage moderators and peers to provide an additional safety net (and additional training data) through manual prioritization. In addition, there are many opportunities to incorporate related work in social media mining to improve the accuracy of the triage algorithm that we hope to explore.

## Appendix

Multimedia Appendix 1 A description of the triage interface, including screenshot.

Multimedia Appendix 2 A description of the triage algorithm, and the features it relies on.

Multimedia Appendix 3 Additional details about how moderators were identified, how replies were tracked for messages, and how the activity histograms were constructed.

## Abbreviations

CLPsych: Computational Linguistics and Clinical Psychology
IQR: interquartile range
OR: odds ratio
