# Supplementary material for: Improving Moderator Responsiveness in Online Peer Support Through Automated Triage
Source: J Med Internet Res. 2019 Apr 26;21(4):e11410. doi: 10.2196/11410 (PMC6658385; doi:10.2196/11410)
Supplement: Multimedia Appendix 1 [file jmir_v21i4e11410_app1.docx]

**Multimedia Appendix 1: The triage interface**

The triage interface consists of a pair of widgets that are injected directly into the forum website (see Figure 1). These widgets check with the forum’s authentication system, and only reveal themselves to users who have been given moderator privileges.

| 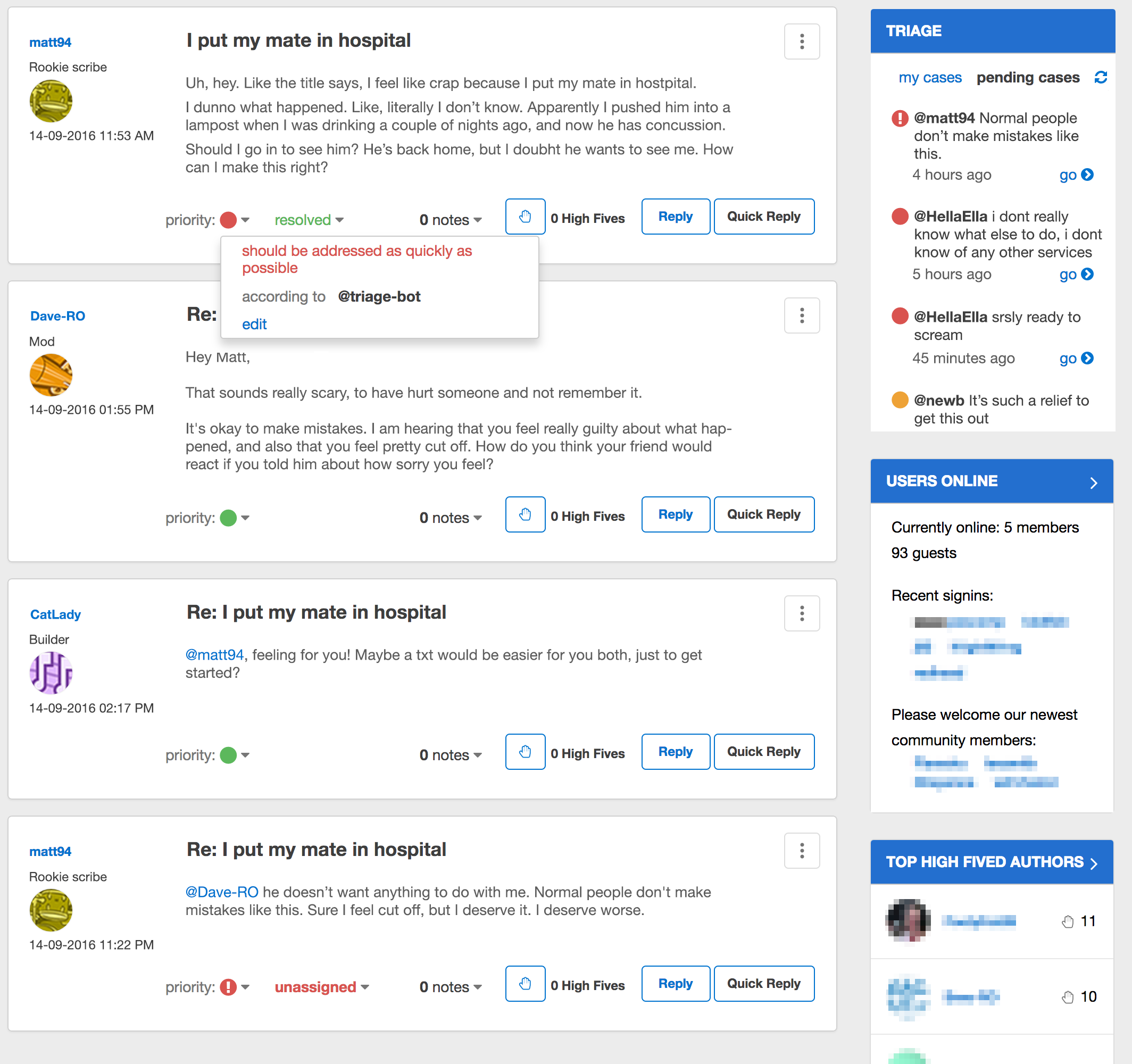 |
| --- |
| Figure 1: A (fictional) forum thread, with triage widgets visible |

The first of the two widgets is injected below every forum message. It briefly describes the priority that has been assigned to the message. For example, the first and last messages in the figure have been prioritized as urgent (red and crisis respectively) while the other two have been prioritized as green.

Hovering over the priority indicator reveals further detail, as shown for the first message in Figure 1. This explains that the red label was assigned by *@triage-bot*, which is a forum account that personifies the automated triage algorithm. If the algorithm makes a mistake, the *edit* link allows moderators to correct it.

To the right of the priority indicator is the status indicator. This shows whether the message is resolved and—if not—whether a moderator has taken responsibility for resolving it. Messages are automatically resolved as soon as they receive a response from a moderator, but moderators can hover over the indicator to manually mark messages as resolved (i.e. if the community as rallied around it) or unresolved (i.e. if they require more attention). They can also assign an unresolved message to themselves, to give themselves time to compose a response and encourage other moderators to focus their efforts elsewhere. The status indicator is not shown for green messages, because these messages do not require any action to be taken.

To the right of the status indicator is a menu for accessing notes that have been posted about the message. These notes are only accessible to moderators, and offer a simple, private communication channel to coordinate responses to individual messages.

The second triage widget is the sidebar shown on the right of Figure 1, which is consistently displayed on every page while moderators browse the forum. This provides a list of prioritized (i.e. not green) messages requiring moderators response. Each message is displayed with a priority, the name of its author, how long ago it was posted, a short excerpt, and a link to the message itself. The ordering encourages moderators to respond to posts in order of priority (crisis posts first, then red, then amber). A secondary ordering encourages moderators to address older messages first. By default, this list displays all pending (i.e. unresolved and unassigned) messages, but the “my cases” link can be clicked to show the unresolved messages that the moderator has assigned to themselves. The short excerpts are generated by running each sentence of a message separately through the algorithm described below, and retaining the sentence that is given the highest probability of being written by someone in crisis.

The system described above has the inevitable effect of highlighting content that many would find distressing and potentially triggering. Consequently, it was only ever shown to trained moderators, all of whom had developed their own self-care plans, and receive ongoing training and support from ReachOut.com staff. However, we did deploy a second version of triage to moderators-in-training and long-standing members of the community. This second version does not include the sidebar and does not reveal any priorities assigned to messages, and consequently does not increase users’ exposure to distressing content. Instead it simply allows users to manually flag any concerning messages that they encounter during their usual browsing. Flagged messages are immediately added to the triage system so they are brought quickly to moderators’ attention, and used as additional training data for the underlying algorithm. In effect, it adds a safety-net of manual annotation, in case the algorithm underestimates the priority of a message.
