# Supplementary material for: Improving Moderator Responsiveness in Online Peer Support Through Automated Triage
Source: J Med Internet Res. 2019 Apr 26;21(4):e11410. doi: 10.2196/11410 (PMC6658385; doi:10.2196/11410)
Supplement: Multimedia Appendix 2 [file jmir_v21i4e11410_app2.docx]

**Multimedia Appendix 2: The triage algorithm**

The algorithm that automatically prioritizes forum messages is a supervised classification system, which represents forum messages as a series of numerical features and—using a training set of manually prioritized messages—builds a model to infer priority labels automatically.

Table 1: Features used by the triage algorithm

| Feature | Representation |
| --- | --- |
| *From message to be prioritized* | |
| Message body | n-grams, with chi-squared feature selection |
| Author rank | one-hot encoding |
| *From first message in the same thread* | |
| Thread subject | n-grams, with chi-squared feature selection |
| Thread starter body | n-grams, with chi-squared feature selection |
| Thread starter author rank | one-hot encoding |
| *From parent message* | |
| Parent message body | n-grams, with chi-squared feature selection |
| Parent message author rank | one-hot encoding |
| *Other* |  |
| Parent board | one-hot encoding |

Table 1 lists the features and feature groups used in the Triage system. It is informed not only by the message being prioritized, but also the context surrounding it. Messages on the forum are organized into *threads*, or individual conversations that begin with a single message and a short subject. These threads are in-turn organized into broad topics of conversation, called *boards*. For example, Figure 1 in Multimedia Appendix 1 shows the start of a (fictional) thread entitled *I put my mate in hospital*, which belongs to a board entitled S*omething’s not right*. Although this figure shows only a simple linear sequence of messages, the forum maintains an internal tree structure in which each message can have any number of children (i.e. replies), but only one parent (the message the author chose to reply to). In the figure, the messages from *@Dave_RO* and *@CatLady* are both children of *@matt94*’s first message, while *@matt94*’s second message is a child of *@Dave_RO*’s.

The body of the message, the subject and body of the first message in the thread, and the body of the parent message provide the text-based features of the system. All text is pre-processed so that hypertext links are replaced with the token *LINK* and images are replaced with *IMG*. Emoticons are replaced with *EMO_POS*, *EMO_NEG* or *EMO_NEUTRAL*, depending on the valence of the emotion they represent, using a gazetteer developed by Shickel and Rashidi [1]. Mentions of forum users are replaced with a token *USER_RANK* that identifies the user's rank in the forum (e.g. *Rookie*, *Uber contributor*). The remaining text is stripped of all HTML markup and then translated into a feature vector using n-grams, TF×IDF (term frequency × inverse document frequency) weighting, and chi-square feature selection to reduce dimensionality.

Rankings are assigned in the ReachOut.com forums to indicate moderator seniority (e.g. *Rookie*, *Uber scribe*), or affiliation (e.g. *Mod Squad*, *Youth Ambassador*). These rankings were used when pre-processing text (described above), and also provide separate features based on the ranking of the authors of the message being prioritized, the author who initiated the thread, and the author of the parent message. Each of these user rank features are represented using one-hot encoding. The final feature of the system is the board it is found within, again represented using one-hot encoding.

It is worth noting that none of these features involve the response or reaction that the message received. While this reaction is likely to be informative (e.g. because people will likely react differently to someone in crisis), we have avoided such features for two reasons. Firstly, we wanted the system to prioritize each message immediately after it is authored, without waiting to analyse the reaction. Secondly, our evaluation of the triage system is based on its ability to influence how moderators respond to forum messages, and we wanted to avoid any cross-contamination between the data used to inform the algorithm and the data used to evaluate it.

The system is agnostic about which underlying algorithm is used to learn the underlying model that translates these features into priorities. The results reported in this paper were obtained after tuning via repeated cross-fold validation over the CLPsych 2016 [2] training set. This determined that the best performing classification algorithm was Support Vector Machine, which was best tuned with the RBF kernel (C=1, gamma=0.125). The algorithm also performed best when including all 1-3 grams from the message body, but using the chi-squared metric to limit thread subject and thread starter body features to 500 and 3000 features respectively, and the parent body features to 1000.

## References

[1] Shickel B, Rashidi P. Automatic Triage of Mental Health Forum Posts. Proceedings of Computational Linguistics and Clinical Psychology; 2016:188–192. San Diego, CA.

[2] Milne DN, Pink G, Hachey B, Calvo RA. CLPsych 2016 Shared Task: Triaging content in online peer-support forums. Proceedings of Computational Linguistics and Clinical Psychology; 2016:118–127. San Diego, CA.
