# Supplementary material for: Improving Moderator Responsiveness in Online Peer Support Through Automated Triage
Source: J Med Internet Res. 2019 Apr 26;21(4):e11410. doi: 10.2196/11410 (PMC6658385; doi:10.2196/11410)
Supplement: Multimedia Appendix 3 [file jmir_v21i4e11410_app3.docx]

**Multimedia Appendix 3: Tracking authors, replies and forum activity levels**

This section provides additional detail about (a) how authors were identified, to separate messages authored by moderators and peers, (b) how replies to messages were tracked, and (c) how general activity levels in the forum were measured for inclusion in the regression analysis. All of these details were extracted directly from the ReachOut.com's forum, which is hosted on the Lithium platform.

**Identifying author roles**

The study is concerned with the behaviour of moderators when they respond to messages posted by ordinary community members. Consequently, it is important to accurately identify the role of the author of each message. This is achieved using an audit log of roles that were granted to members of the forum. Table 1 lists the roles used to identify whether a forum member is a moderator, a trainee, or some other affiliate of the forum. Moderators include volunteers (young peers who are recruited and trained each year), staff (who are employed by ReachOut), and external contractors (who provide an after-hours safety net). Trainees are individuals who are receiving online training to become volunteer moderators, but have not yet graduated. Other affiliates are individuals who have been invited to contribute to the forum because of their expertise or prior experience.

| Table 1: Roles used to identify moderators, trainees and affiliates | |
| --- | --- |
| User type | Associated roles |
| Volunteer moderator | mod squad, cheer squad, post-mod |
| Staff moderator | moderator, staff, administrator, community manager, reachout.com crew |
| Externally contracted moderator | on the line, ontheline |
| Trainee moderator | mod in training, in training, work experience |
| Affiliate | youth ambassador, youth editorial board, special guest contributor, researcher, ro film crew, category expert |

This study considered only replies made by moderators, to messages authored by ordinary community members. All messages made by trainees and affiliates were ignored, as were messages made by moderators unless in response to a message from an ordinary community member.

**Tracking message replies**

Unfortunately, tracking replies in a forum is not an exact process. Although the Lithium platform allows messages to be replied to directly and keeps track of the full hierarchy of replies, users often ignore this functionality and instead reply indirectly by posting a new message later in the same thread. Additionally, threads can persist for a long time and host multiple unconnected conversations. Consequently, two messages are not necessarily connected even though they belong to the same thread. To further complicate matters, a response might be crafted to reply to multiple messages.

To gather all replies to a message we first gather all direct replies to it (i.e. all immediate children in the reply hierarchy). The direct replies are supplemented by working forward through the thread to gather every message that mentions the username of the original author. Replies will be gathered in this way for up to three working days after the original message, or until two more messages from the original author are encountered; whichever comes first. The triage system described previously uses this same algorithm to decide whether a post can be automatically marked as resolved (i.e. if it has received a reply from a moderator).

**Measuring forum activity levels**

When measuring the impact of the triage system on moderator behaviour, it was important to also account for other factors that might also have an impact. In this study, the underlying covariate we aimed to account for was the workload placed on moderators, which is influenced by how busy the forum is at any given time, and how many moderators are online and able to share the load.

Both of these factors are likely to follow long and short-term trends. In the long term, we can expect a healthy forum to get gradually busier over time and for moderators to be most active immediately after an influx of new volunteers (which occurs roughly yearly). In the short-term, we can expect the forum to be busier and moderators to be more active at times of day and days of week when access to the forum is more convenient.

|  |  |
| --- | --- |
| a) Number of messages posted by moderators | b) Number of messages posted by ordinary community members |
|  |  |
| c) Number of moderators posting at least one message | d) Number of ordinary community members posting at least one message |
| Figure 1: Example histograms of forum activity surrounding the creation of a message | |

To account for these patterns, we built histograms that capture activity levels of moderators and ordinary community members at and around the time that each message was posted. For each author type we separately recorded both the number of messages created, and the number of unique accounts that posted at least one message. This resulted in four histograms, each of which consisting of 5 hour-long periods starting 2 hours prior to the message being created, and ending 3 hours afterwards.

Figure 1 provides examples of these four histograms for a message that occurred in the early evening. In this example, the forum was not busy prior to the message being posted, so there is unlikely to be a backlog of content for moderators to address. However, moderators were not very active at the time the message was posted, so the message is unlikely to receive an immediate response. It is more likely that it received a response an hour later, when there is a spike in moderator activity.
